# TRIB1 promotes colorectal cancer cell migration and invasion through activation MMP-2 via FAK/Src and ERK pathways

**DOI:** 10.18632/oncotarget.18201

**Published:** 2017-05-25

**Authors:** Yuhui Wang, Nan Wu, Bo Pang, Dandan Tong, Donglin Sun, Haiming Sun, Chunyu Zhang, Wenjing Sun, Xiangning Meng, Jing Bai, Feng Chen, Jingshu Geng, Songbin Fu, Yan Jin

**Affiliations:** ^1^ Laboratory of Medical Genetics, Harbin Medical University, Harbin, China; ^2^ Department of Pathology, Harbin Medical University, Harbin, China; ^3^ Department of Pathology, Third Affiliated Clinical Hospital, Harbin Medical University, Harbin, China; ^4^ Key Laboratory of Medical Genetics, Harbin Medical University, Heilongjiang Higher Education Institutions, Harbin, China

**Keywords:** TRIB1, colorectal carcinoma, migration, invasion, MMP-2

## Abstract

Colorectal cancer (CRC) is the third most common cancer in the world and distant metastasis is the leading cause of death among CRC patients. However, the underlying mechanisms of distant metastasis remain largely unknown. Amplification of 8q24 is a common chromosomal abnormality in CRC. In the present study, a putative oncogene at 8q24, TRIB1, was characterized for its role in CRC metastasis and underlying molecular mechanisms. Higher expression of TRIB1 protein was detected in 58/83 (69.9%) of CRC tissues, compared with adjacent non-tumor tissues. Moreover, the expression of TRIB1 was significantly associated with distant metastasis (*P*=0.043) and advanced staging (*P*=0.008) in CRC tissues. TRIB1 overexpression was also correlated with poor prognosis in CRC patients as analyzed in PrognoScan database. In addition, elevated expression of TRIB1 promoted CRC cell motility and adhesive ability, while silencing of TRIB1 reduced those effects. Further study revealed that TRIB1-mediated migration and invasion of CRC cells required up-regulation of MMP-2 through the activation of FAK/Src and ERK pathway. Collectively, the results suggest that TRIB1 promotes CRC cell motility by activation MMP-2 via the FAK/Src and ERK pathways. It may provide a potential diagnostic and therapeutic target for CRC.

## INTRODUCTION

Colorectal cancer (CRC) is the second most common cancer in females and the third in males, with about 1.4 million cases and 693,900 deaths occurring globally in 2012 [1]. The major cause of death in CRC patients is distant metastasis. Tumors that are confined within the wall of colon (stages I and II) are curable by surgical excision, and approximately 73% of CRC cases with lymph node metastasis (stage III) are curable by surgery combined with adjuvant chemotherapy. However, patients with distant metastasis are usually incurable, although survival has been improved by recent advances in chemotherapy [2]. Therefore, to understand the underlying molecular mechanisms involved in the development of metastasis is extremely important.

Double minute chromosomes (DMs) are small, paired, acentric extrachromosomal structures. They are considered to be hallmarks of gene amplification [3]. Many genes amplified on DMs have been proved to be oncogenes and play an important role in tumorigenesis and cancer progression [4]. To explore the molecular characteristics of DM-carried genes in tumor cells, our colleagues performed Affymetrix SNP Array 6.0 analyses and identified the amplification regions in CRC cell line NCI-H716, which is known to contain lots of DMs [5]. The results showed that 8q24 was the main amplified region and Tribbles pseudokinase 1 (TRIB1) was one of the genes located in it. Amplification of 8q24 is a common chromosomal abnormality in CRC [6] and some other cancers including acute myeloid leukemia (AML) [7, 8], prostate cancer [9], gastric cancer [10], malignant mesothelioma [11], esophageal carcinoma [12] and ovarian cancer [13]. These suggest that 8q24 is significantly associated with human cancers and this region may carry oncogenes related to tumorigenesis and/or progression. TRIB1, which belongs to the Trib family, is classified as pseudokinase and has important roles in many cellular processes [14]. TRIB1 has been identified as an oncogene in AML [15] and prostate cancer [16]. However, its role in CRC is still unclear.

In the present study, we first evaluated the relationship between expression level of TRIB1 and clinicopathological features in CRC tissues. Moreover, the effect of TRIB1 on cell migration and invasion was investigated using a series of assays and the underlying mechanisms were explored.

## RESULTS

### TRIB1 is frequently amplified and overexpressed in CRC tissues

**Figure 1:**
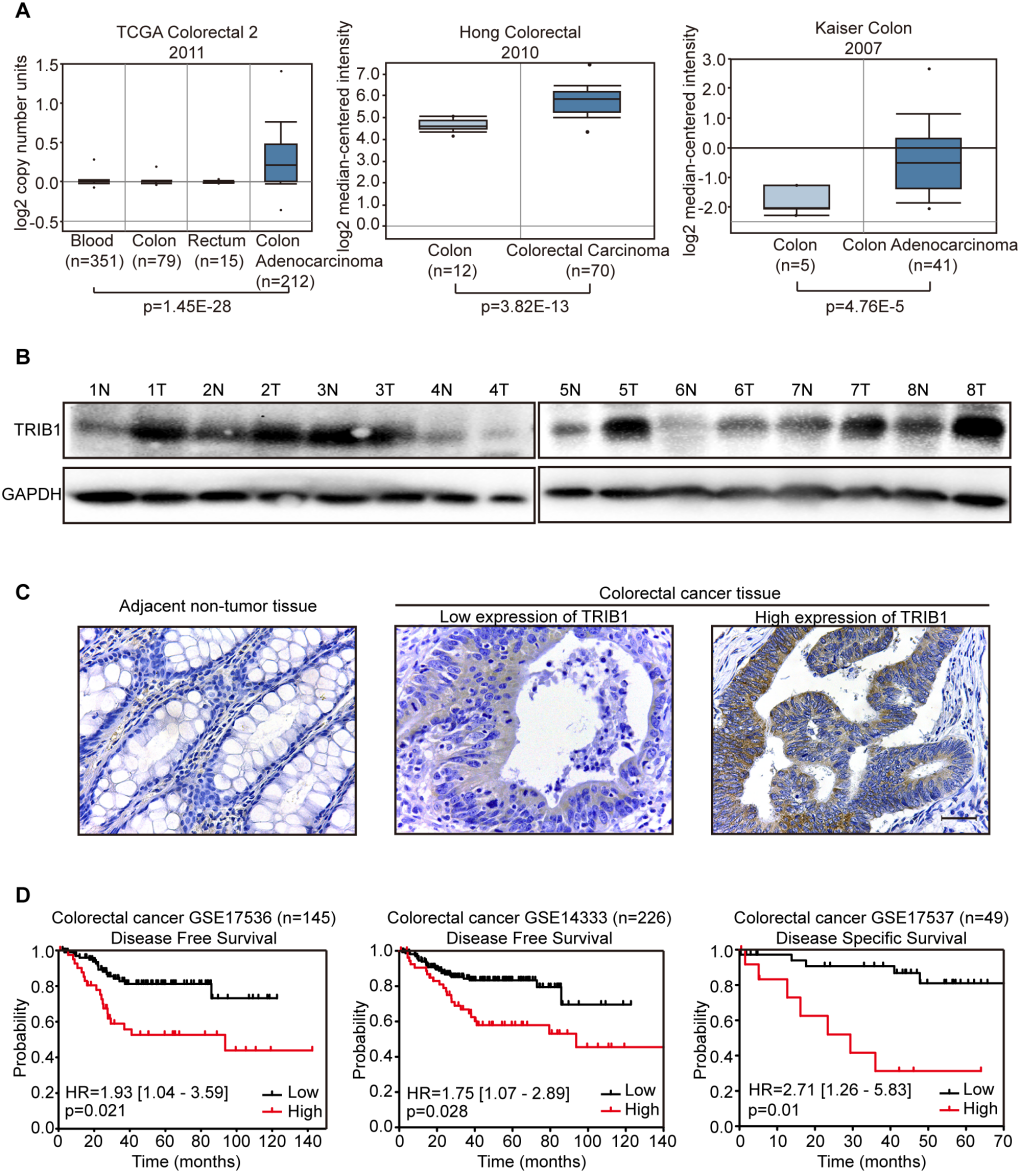
TRIB1 is frequently amplified and overexpressed in human colorectal cancer (CRC) tissues **(A)** TRIB1 copy number and mRNA levels in CRC and non-tumor tissues are shown as box plots. Data from TCGA and two microarray expression datasets was obtained from the Oncomine database. **(B)** The levels of TRIB1 protein in CRC specimens were detected by western blot analysis, T, tumor tissue; N, normal tissue. GAPDH was used as loading control. **(C)** Representative photographs of TRIB1 expression in CRC tissue and adjacent normal tissue examined by IHC (magnification ×400) are presented. Scale bars = 400μm. **(D)** Kaplan–Meier curves indicating the disease free survival or disease specific survival are shown. The expression levels of TRIB1 and clinical information were obtained from publicly available databases (GSE17536, GSE14333 and GSE17537) and analyzed using PrognoScan, which is a great tool for evaluating the relationship between gene expression and prognosis.

Firstly, the copy number of TRIB1 was analyzed in TCGA database using Oncomine. Data showed that the copy number of TRIB1 in CRC tissues was significantly increased compared with that in normal blood, colon or rectum tissues (Figure 1A left). The mRNA expression levels of TRIB1 were also found elevated in CRC tissues compared with normal colon tissues as analyzed in two microarray expression studies from Oncomine (GSE9348 and GSE5206) (Figure 1A middle and right). To explore its expression in CRC, we detected TRIB1 protein level in 8 pairs of CRC tumor and surrounding non-tumor tissues by western blotting. The results showed that 6 out of 8 (75%) CRC tissues had elevated TRIB1 expression, when compared with paired non-tumor tissues (Figure 1B). In addition, IHC was used to examine TRIB1 expression in 75 pairs of CRC tissues and matched adjacent non-tumor tissues. Overexpression of TRIB1 was detected in 52/75 (69.3%, *P*<0.05, Wilcoxon's signed-rank tests) of CRC as compared with adjacent non-tumor tissues (Figure 1C). Collectively, these results indicate that TRIB1 is frequently amplified and overexpressed in human CRC tissues.

### Clinical significance of TRIB1 overexpression in CRC

We further evaluated the correlation of TRIB1 overexpression with clinicopathological features using IHC data. The results showed that overexpression of TRIB1 was positively associated with distant metastasis (*P*=0.043) and advanced staging (*P*=0.008) (Table 1). Moreover, the results indicated that TRIB1 overexpression was significantly associated with distant metastasis (*P*=0.002, Supplementary Table 1) in CRC samples from GEO database (GSE17537). To investigate whether overexpression of TRIB1 is correlated with poor prognosis in CRC patients, the PrognoScan database was used. PrognoScan is a huge collection of cancer microarray datasets and clinical information which can be available publicly, and is also a great tool for evaluating the relationship between gene expression and prognosis [17]. Three databases (GSE17536, GSE14333 and GSE17537) from PrognoScan were analyzed. The results show that overexpression of TRIB1 is correlated with poor survival in CRC patients (Figure 1D).

**Table 1:** Correlation between TRIB1 expression and clinicopathological factors in colon cancer

Variable	Cases	TRIB1 expression		*P* value
		High	Low	
Gender				
Female	32	21(65.6%)	11(34.4%)	0.800^a^
Male	43	27(62.8%)	16(37.2%)	
Age(years)				
<63	30	21(70.0%)	9(30.0%)	0.403^a^
≥63	43	26(60.5%)	17(39.5%)	
Lymph node metastasis				
-	39	24(61.5%)	15(38.5%)	0.644^a^
+	36	24(66.7%)	12(33.3%)	
Distant metastasis				
-	66	39(59.1%)	27(40.9%)	0.043^b^*
+	9	9(100.0%)	0(0.0%)	
Differentiation				
1-2	10	8(80.0%)	2(20.0%)	0.436^b^
3	65	40(61.5%)	25(38.5%)	
Staging				
I–III b	60	34(56.7%)	26(43.3%)	0.008^a^*
III c–IV	15	14(93.3%)	1(6.7%)	

^a^Pearson χ2 test; ^b^Yates correction χ2 test; *P<0.05.

### Ectopic TRIB1 expression promotes motility and adhesion of CRC cells

**Figure 2:**
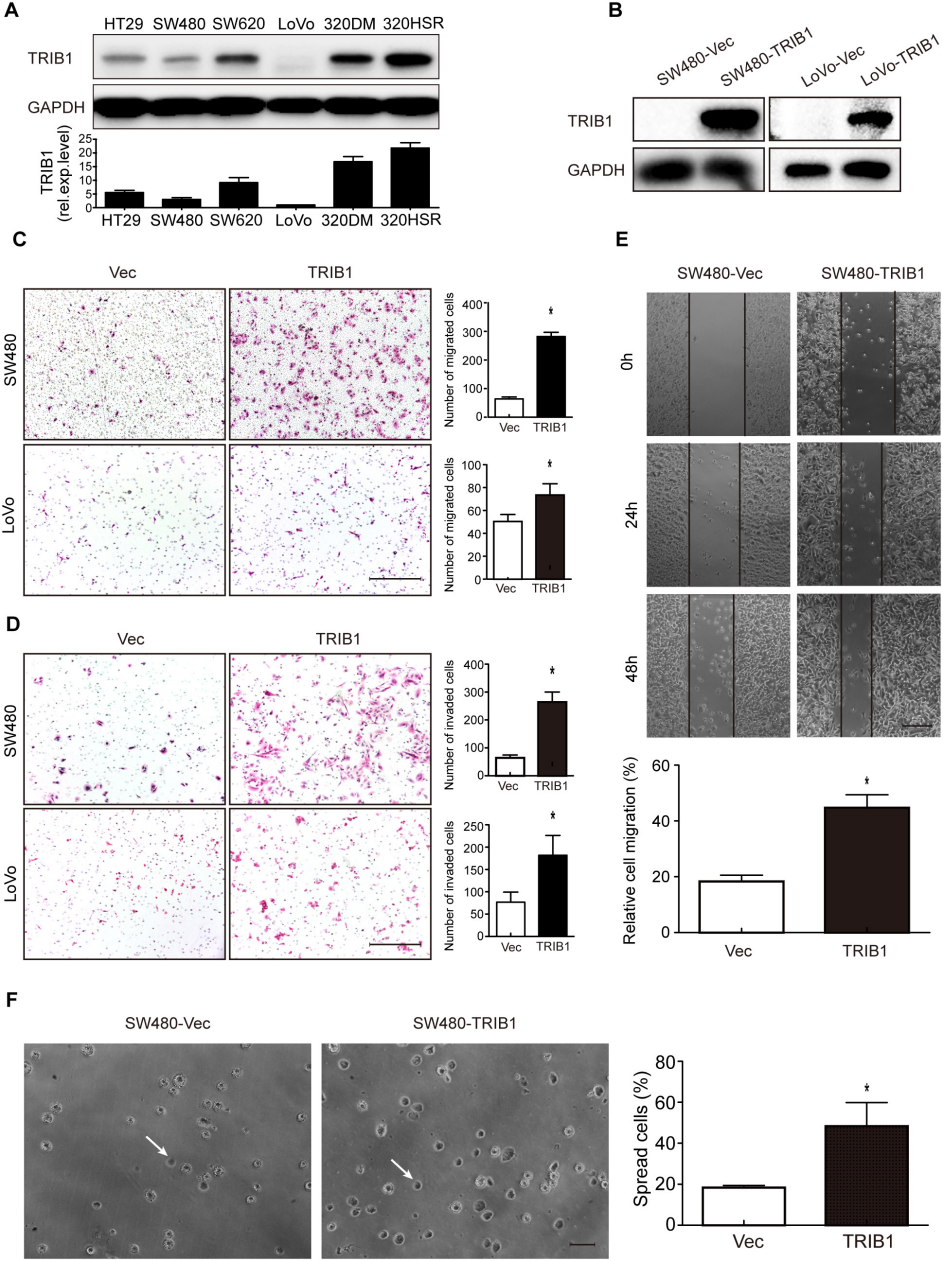
TRIB1 promotes motility and spreading of CRC cells **(A)** Expression levels of TRIB1 in CRC cell lines were measured with western blotting. **(B)** Ectopic TRIB1 expression in the CRC cell lines SW480 and LoVo was examined by western blotting. **(C-D)** Transwell migration (C) and invasion (D) assays were performed to examine cell migration and invasion in SW480 and LoVo cells overexpressing TRIB1. Representative pictures of migrated or invaded cells are displayed in the left panels (magnification ×100) and the results are shown in the right panels. Scale bars = 200μm. Columns indicate the mean ± SD of triplicate experiments (*P<0.01, independent Student's t-test). **(E)** Wound-healing assay was used to investigate cell motility of SW480 cells overexpressed with TRIB1. Cells transfected with empty vectors were used as control. Representative images are shown in the upper panel (magnification ×40). Scale bars = 200μm. Results are summarized as mean ± SD of triplicate experiments (*P<0.01, independent Student's t-test) and shown in the bottom panel. **(F)** Spreading of SW480-TRIB1 or SW480-Vec cells onto 96-well plates coated with 5μg/ml FN was assessed at 45 minutes after seeding. The results showed that SW480-TRIB1 cells (48.3%) significantly promoted cell adhesion compared with control cells (18.3%). (F) Representative photographs were shown in the left panel (magnification ×200). Scale bars = 400μm. White arrows indicate a spreading cell. Results are presented as mean ± SD of triplicate experiments (*P<0.01, independent Student's t-test) and shown in the right panel.

As the clinical correlation analysis revealed that TRIB1 overexpression was positively associated with CRC metastasis, it is necessary to investigate the effect of TRIB1 on cell motility. We first detected expression levels of TRIB1 in a series of CRC cell lines (HT29, SW480, SW620, Lovo, COLO320DM and COLO320HSR) by western blotting. SW480 and LoVo cell lines, which showed the lowest TRIB1 expression (Figure 2A), were chosen for the subsequent overexpression studies. The pEGFP-C1-TRIB1 was stably and transiently transfected into SW480 and LoVo cells, respectively. Cells transfected with pEGFP-C1 empty vectors were used as negative control (Figure 2B).

Wound-healing, transwell migration and invasion assays were used to characterize the role of TRIB1 in cell migration and invasion. The transwell migration assay demonstrated that elevated expression levels of TRIB1 remarkably enhanced the migratory ability of SW480 and LoVo cells (Figure 2C). In the invasion assay, cells transfected with TRIB1 gained a higher rate of invasion compared with control cells (Figure 2D). In addition, the wound-healing assay revealed that cell migration was significantly faster in SW480-TRIB1 cells than in SW480-Vec cells (Figure 2E). As adhesion is also an essential feature of metastasis, cell spreading assay was performed to investigate the role of TRIB1 in CRC cell adhesion to extracellular matrix (ECM). The results showed that TRIB1 overexpression in SW480 cells remarkably promoted cell adhesion on FN-coated plates compared with control cells (Figure 2F).

**Figure 3:**
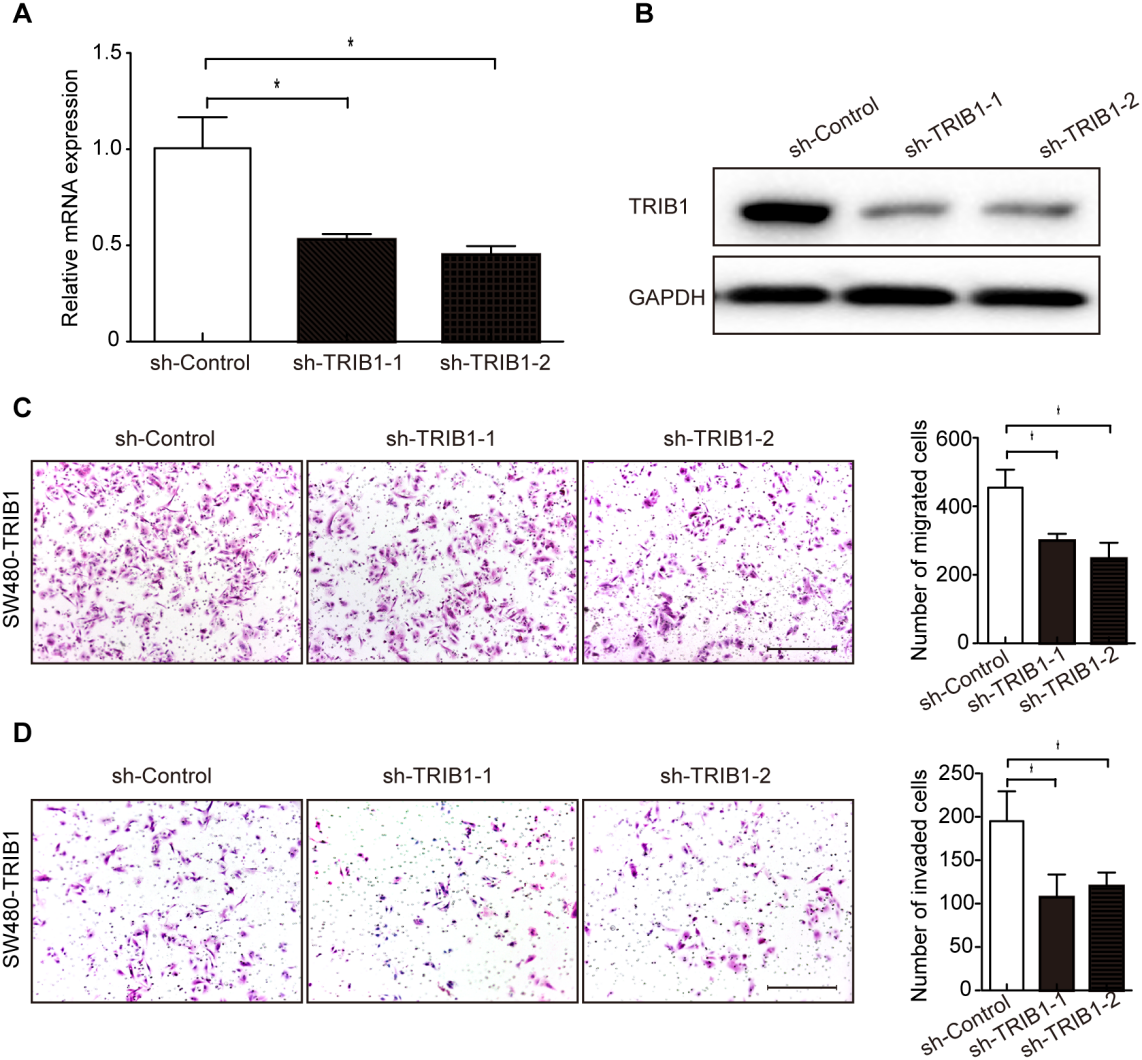
Silencing of TRIB1 reduces cell migration and invasion **(A-B)** In SW480-TRIB1 cells, the efficiency of sh-TRIB1-1 or sh-TRIB1-2 interference was confirmed by qRT-PCR (A) and western blot analysis (B). Corresponding shRNA (sh-Control) was used as negative control and GAPDH was used as loading control. In qRT-PCR results, the expression of sh-Control was set as 1. Data are presented as mean ± SD of triplicate experiments (*P<0.01, independent Student's t-test). **(C-D)** Cell migration (C) and invasion (D) were detected using transwell migration chamber and Matrigel invasion chamber, respectively. Examples of migrated or invaded cells are displayed in the left panels (magnification ×100). Scale bars = 200μm. Results are summarized as mean ± SD of triplicate experiments (*P<0.01, independent Student's t-test) and shown in the right panels.

To confirm the ability of promoting cell motility of TRIB1, we next performed gene-silencing using two specific shRNAs against TRIB1 (sh-TRIB1-1 and sh-TRIB1-2). Both shRNAs could effectively knock down TRIB1 in SW480-TRIB1 (Figure 3A-3B) and COLO320HSR cells (Supplementary Figure 1A). The results showed that silencing of TRIB1 led to an obvious reduction of cell migration and invasion in SW480-TRIB1 (Figure 3C-3D) and COLO320HSR cells using transwell migration and invasion assays (Supplementary Figure 1B-1C). In summary, these results demonstrated that overexpression of TRIB1 is vital for the invasion and metastasis of CRC cells.

### TRIB1 up-regulates MMP-2 through the activation of FAK/Src and ERK pathways

**Figure 4:**
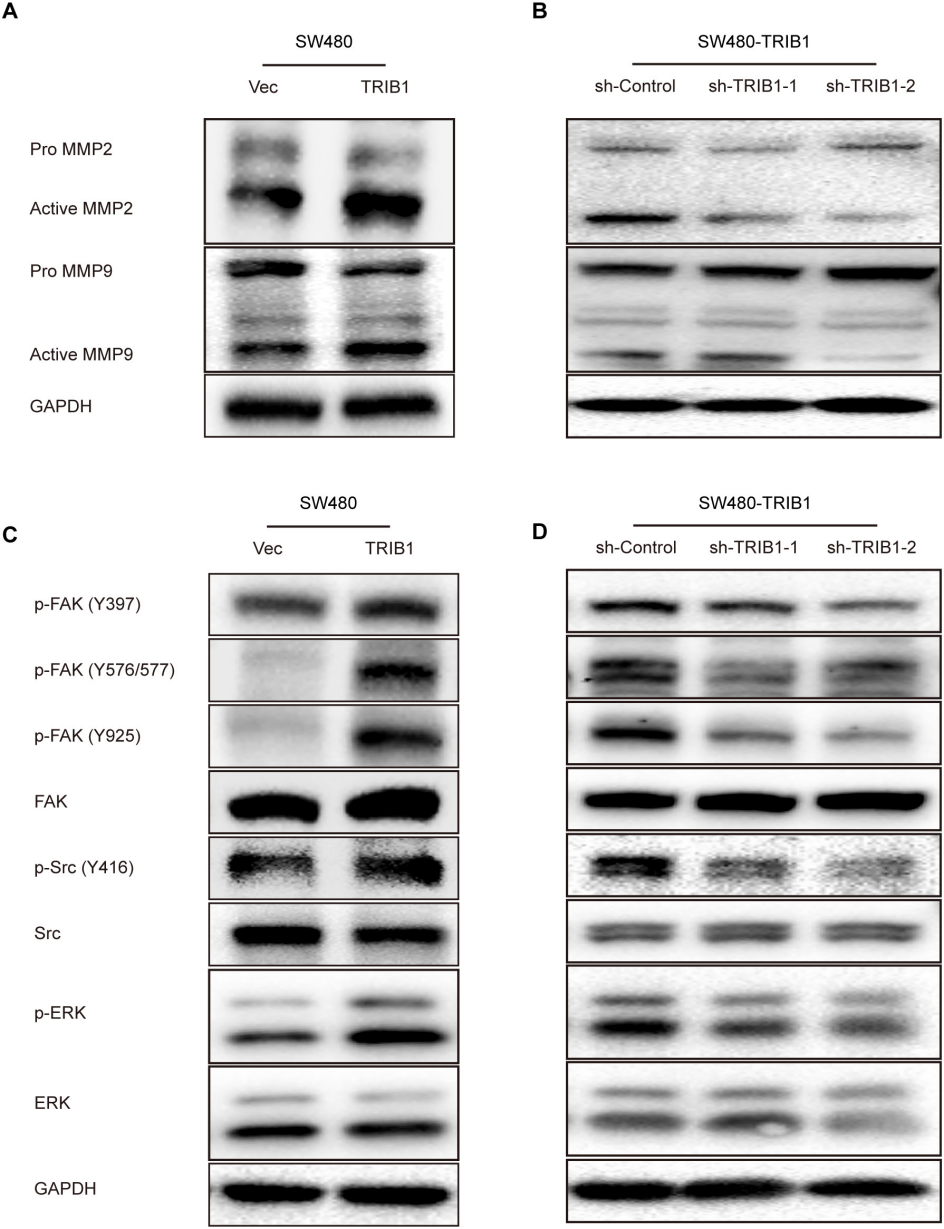
TRIB1 promotes MMP-2 and MMP-9 expression and activates FAK and ERK signaling **(A)** Western blotting assay for MMP-2 and MMP-9 was used in SW480 cells with or without TRIB1. **(B)** SW480-TRIB1 cells were transfected with TRIB1 shRNA or control shRNA. MMP-2 and MMP-9 expression were analyzed by western blotting 72h after transfection. **(C)** Western blotting assay for FAK, Src and ERK was used in SW480 cells with or without TRIB1. **(D)** SW480-TRIB1 cells were transfected with TRIB1 shRNA or control shRNA. FAK, Src and ERK activation were analyzed by western blotting 72h after transfection.

The MMPs family is commonly found to participate in ECM component degradation, which is involved in cell invasion. MMP-2 and MMP-9 are the two most widely studied members of this family. To determine how TRIB1 facilitates CRC cell motility, the expression of the two proteolytic enzymes were analyzed by western blotting in SW480 cells after TRIB1 transfection. Results indicated that MMP-2 and MMP-9 were both up-regulated after TRIB1 transfection(Figure 4A). In addition, RNA interference used to knock down TRIB1 in TRIB1-overexpressed SW480 cells confirmed the results (Figure 4B).

Changes in cell motility and adhesion to ECM suggested that the FAK pathway might be involved. Early studies demonstrated that TRIB1 was associated with MAPK signaling pathway. Therefore, to obtain further insights into the underlying mechanisms by which TRIB1 activates MMP-2 and MMP-9 expression, we detected tyrosine phosphorylation of several important kinases, including FAK, Src and ERK, using western blotting. As shown in Figure 4C, the ectopic expression of TRIB1 in SW480 cells increased the phosphorylation of FAK (Y397), FAK (Y576/577), FAK (Y925), Src (Y416) and ERK proteins. RNA interference was also used to confirm these results (Figure 4D).

**Figure 5:**
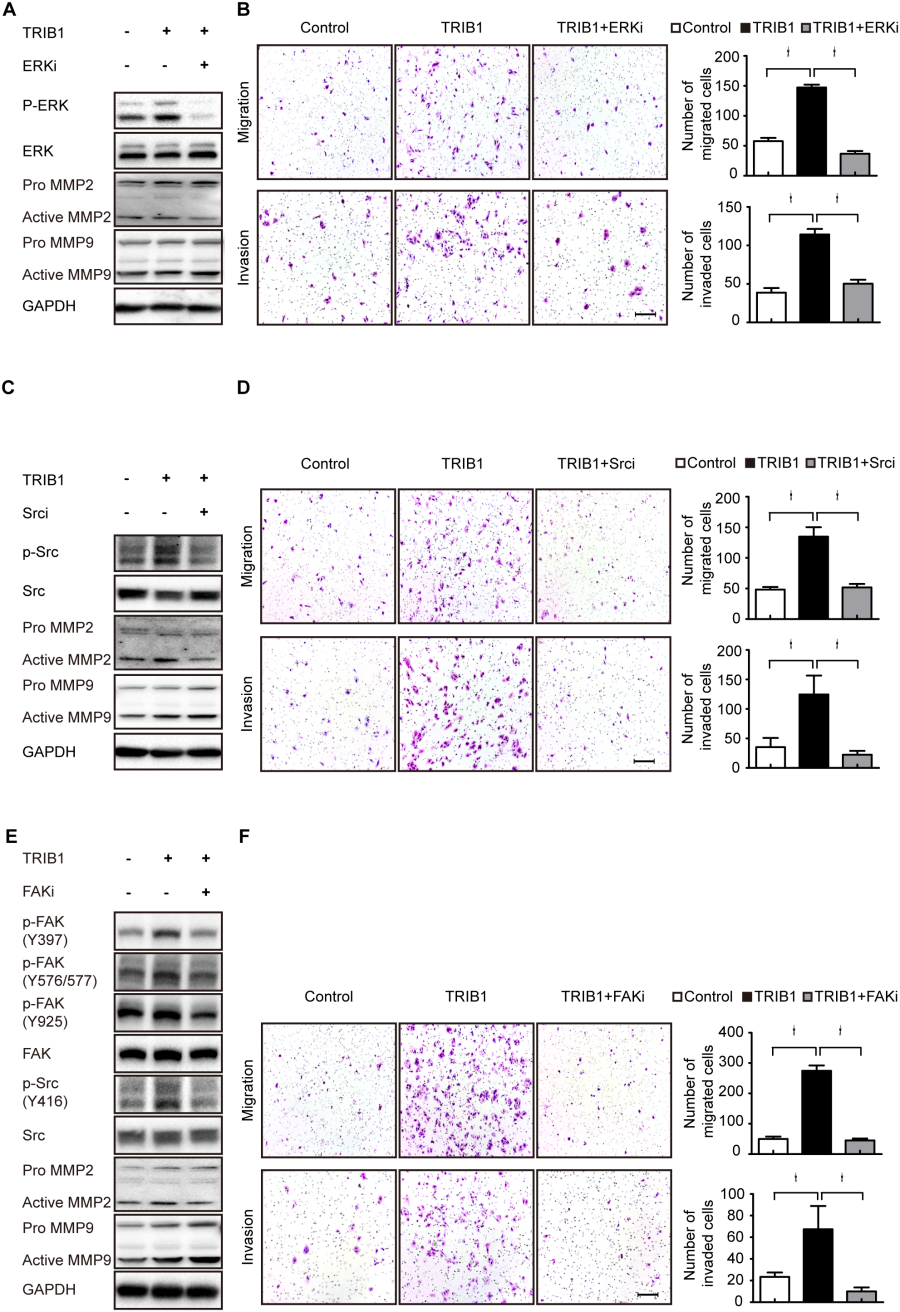
TRIB1 up-regulates MMP-2 expression through FAK/Src and ERK pathways **(A)** SW480-TRIB1 cells were treated with the ERK inhibitor (SCH772984) for 24h, and then ERK activation and MMP-2/MMP-9 expression were detected by western blotting. SW480-Vec cells were used as negative control. **(B)** Transwell assay for migration and invasion were performed using SW480-TRIB1 cells treated with or without SCH772984 for 24h. SW480-Vec cells were used as negative control. **(C)** SW480-TRIB1 cells were treated with the Src inhibitor (PP2) for 24h, and then Src activation and MMP-2/MMP-9 expression were detected by western blotting. **(D)** Transwell assay for migration and invasion were performed using SW480-TRIB1 cells treated with or without PP2 for 24h. **(E)** SW480-TRIB1 cells were treated with the FAK inhibitor (PF562271) for 24h, and then FAK/Src activation and MMP-2/MMP-9 expression were detected by western blotting. **(F)** Transwell assay for migration and invasion were performed using SW480-TRIB1 cells treated with or without PF562271 for 24h.

Further studies showed that ERK inhibitor could down-regulate MMP-2 rather than MMP-9 expression in TRIB1-transfected SW480 cells and decreased their migratory and invasive abilities (Figure 5A-5B). Moreover, FAK inhibitor and Src inhibitor used in TRIB1-expression cells remarkably inhibited MMP-2 expression and reduced the migratory and invasive abilities of CRC cells (Figure 5C-5F). These results demonstrate that TRIB1 activates the FAK/Src and ERK pathways to up-regulate MMP-2 expression and facilitate CRC cell motility.

## DISCUSSION

Previously, our colleagues identified that 8q24 was amplified through DMs in CRC cells [5]. Amplification of 8q24 is one of the most common chromosomal abnormality in CRC [6] and in other cancers such as AML [7, 8], prostate cancer [9] and ovarian cancer [13]. TRIB1, which is located on chromosome 8q24, has been reported to contribute in many kinds of cancers [15, 16], but its role in CRC has not been described.

In the present study, Oncomine database analysis reveals that TRIB1 is frequently amplified and overexpressed in CRC. In addition, we examine the protein expression of TRIB1 in a series of CRC and adjacent non-tumor tissues using western blotting and IHC. Overexpression of TRIB1 is detected in 69.9% (n=83) of CRC tissues, which is significantly correlated with distant metastasis (*P*=0.043) and advanced staging (*P*=0.008). Furthermore, overexpression of TRIB1 is significantly associated with poor prognosis (*P*<0.05) in CRC as analyzed in several databases from PrognoScan. These data strongly suggests that TRIB1 plays a vital role in the progression of CRC.

A series of functional experiments, such as wound-healing assay, transwell migration and invasion assay, demonstrate that ectopic expression of TRIB1 in CRC cell lines remarkably promotes cell migration and invasion. These effects are effectively suppressed when TRIB1 is knocked down with shRNAs. In addition, overexpression of TRIB1 is found to enhance CRC cell spreading. These results indicate that TRIB1 overexpression facilitates CRC cell metastasis and invasiveness.

A crucial step in CRC invasion is the degradation of basement membrane, which is catalyzed by proteolytic enzymes, like MMPs [18]. In this study, up-regulated expression of MMP-2 and MMP-9 are observed by western blotting analysis. Lots of researches have indicated that MMP-2 and MMP-9 could be up-regulated through the activation of FAK and MAPK axis [18–20].

Early studies have indicated that TRIB1 exerts its oncogenic function in AML through the MEK/ERK pathway [21]. Our data indicates that TRIB1 can enhance the phosphorylation of ERK in CRC cells. Moreover, a novel discovery of our study is that TRIB1 expression is correlated with the activation of FAK signaling. Activation of FAK is observed in a range of cancer cells, and it is associated with the progression and distant metastasis of human CRC [22–24]. Phosphorylation of FAK and other focal adhesion complex-associated proteins (like Src) are needed for the formation of focal adhesion, cell motility and invasion [25]. Our results demonstrate that TRIB1 can phosphorylate FAK and Src tyrosine, which involve Tyr-397, Tyr-576/577, Tyr-925 of FAK and Tyr-416 of Src. Furthermore, we found that up-regulation of MMP-2 was dependent on the activation of FAK/Src and ERK pathways, and it was required in the TRIB1-mediated migration and invasion of CRC cells.

In summary, our study reveals that TRIB1 promotes CRC cell migration and invasion by up-regulating the expression of MMP-2 via the activation of FAK/Src and ERK pathways, knockdown of TRIB1 expression in CRC cells abolishes these effects. The data will support TRIB1 as a vital biomarker for CRC diagnosis. We also propose that the interruption of the TRIB1-FAK-Src-MMP-2 and/or TRIB1-ERK-MMP-2 pathways might be a novel therapeutic method for controlling CRC metastasis.

## MATERIALS AND METHODS

### Cell lines and cell culture

The human CRC cell lines SW480, SW620, COLO320DM and COLO320HSR were purchased from the American Type Culture Collection (ATCC, Manassas, VA). Another two cell lines LoVo and HT29 were obtained from the Type Culture Collection of the Chinese Academy of Sciences (Shanghai, China). All of them were cultured as methods described in ATCC.

### Clinical samples and TMA

Sixteen frozen tissues including CRC (n=8) and paired non-tumor tissues (n=8) were obtained from the Third Affiliated Hospital (Harbin Medical University, Harbin, China). The tissue microarray (TMA) containing 75 pairs of human CRC and matched adjacent non-tumor tissues was purchased from Outdo Biotech CO. Ltd. (Shanghai, China).

### Immunohistochemistry (IHC)

PowerVision^™^ Two-Step Histostaining Reagent (Zhongshan Golden Bridge, Beijing, China) was used in immunohistochemical staining. After deparaffinization and rehydration, tissues were incubated in 3% H_2_O_2_ for 10 min at room temperature. For antigen retrieval, the slides were placed in 10mM citrate buffer (PH 6.0) using a microwave oven. Then rabbit primary antibody against human TRIB1 (1:200, Abcam, Cambridge, MA) was added to the tissues and incubated overnight at 4°C. Then the slides were incubated with goat anti-rabbit IgG at 37°C for 30 min. Next, 3’-diaminobenzidine was used for the color reaction. After counterstaining for nuclei with hematoxylin, the slides were dehydrated, cleared and mounted. As to negative controls, the same experimental conditions were used, except that they were incubated overnight without the primary antibody. Staining intensity was scored from 0 to 3 and evaluated by three pathologists.

### Western blotting

Proteins extracted from cell lysates and tissue lysates were separated by 10% SDS-PAGE and transferred onto PVDF membrane. The following primary antibodies were used: GAPDH (Kangchen Bio-tech, Shanghai, China), TRIB1 (Abcam), MMP2 (Proteintech), MMP9 (Proteintech), Phospho-FAK (Tyr397), Phospho-FAK (Tyr576/577), Phospho-FAK (Tyr925), FAK, Phospho-Src (Tyr416), Src, ERK1/2, Phospho-ERK1/2 (All from Cell Signaling Technology, USA). ERK inhibitor (SCH772984), Src inhibitor (PP2) and FAK inhibitor (PF562271) were obtained from Selleck Chemicals (Houston, TX, USA).

### Quantitative real-time polymerase chain reaction (qRT-PCR)

Total RNA was extracted with the High Pure RNA Isolation Kit (Roche, Basel-Stadt, Switzerland) following the manufacturer's recommended protocol. Reverse transcription was performed using the PrimeScript^®^ RT Reagent Kit Perfect Real Time (Takara, Dalian, China). Quantitative RT-PCR was undertaken using the LightCycler^®^ 480 SYBR Green I Master (Roche) and the assay was performed on a CFX96™ Real-Time System (BIO-RAD). For TRIB1 mRNA analysis, GAPDH was used as an internal control. Primer sequences were as follows:

TRIB1 forward 5′-CGGAGGAGAGAACCCAG CTT-3′,

reverse 5′-GCAGCCTTTCCGGAGTAGGT-3′;

GAPDH forward 5′-ATCACTGCCACCCAGAA GAC-3′,

reverse 5′-TTTCTAGACGGCAGGTCAGG-3′.

The relative expression of TRIB1 was calculated according to the 2^-ΔΔCt^ method.

### Plasmid constructs and transfection

Full-length human TRIB1 complementary DNA (cDNA) was amplified by PCR and cloned into pEGFP-C1 expression vector, which was kindly provided by Dr. ZH Zhong (Department of Microbiology, Harbin Medical University, Harbin, China). Then it was transfected into SW480 and LoVo cells using Lipofectamine 2000 (Invitrogen) according to the manufacturer's instructions. Cells transfected with pEGFP-C1 vector were used as controls. Stable TRIB1-expressing SW480 clones were selected by G418 (Calbiochem, Darmstadt, Germany) at a concentration of 1000 μg/mL. The short hairpin RNAs (sh-TRIB1) were constructed as described previously [5] and transiently transfected into SW480-TRIB1 cell line using Lipofectamine 2000. Cells transfected with empty vector were used as controls.

### Wound-healing assay

SW480 cells were cultured on a six-well plate until confluence and then scratched with a 10μl pipette tip. After washed with PBS three times to remove debris, cells were cultured till 48h. Images were captured at 0, 24, and 48h after scrathing to evaluate the rate of wound healing.

### Cell migration and invasion assays

Migration and invasion assays were performed using Corning chambers (Corning, MA, USA) with Matrigel (for invasion assay) or without Matrigel (for migration assay). The cells were suspended in serum-free media and were seeded on the upper chamber. Culture medium containing 20% FBS was placed in the lower chamber. After incubation for 24h or 48h at 37°C, the remaining Matrigel and cells on the upper surface were gently removed with a cotton swab. The cells on the lower surface of the membrane were fixed with methanol and stained with hematoxylin and eosin. Cells in five randomly chosen visual fields (100× magnification) were counted. The experiment was independently repeated three times.

### Cell spreading assay

SW480 cells were resuspended in L15 + 10% FBS and seeded on 96-well plates coated with 5μg/ml fibronectin (FN). The plates were kept at 37°C under 5% CO_2_ for 45 minutes. Then cells were fixed with 50% glutaraldehyde for 30 minutes. Photographs were taken at 200× magnification using a phase contrast microscope (Nikon, Japan). Number of spread cells was counted.

### Statistical analyses

SPSS 19.0 (IBM; Armonk, NY, USA) was used for statistical analysis. The expression differences between CRC and adjacent non-tumor tissues in TMA were analyzed by Wilcoxon's signed-rank tests. The Pearson χ2 test or Yates correction χ2 test were used to analyze the correlations of TRIB1 expression with different clinicopathological parameters. Other data were expressed as the means ± standard deviation (SD), and two-tailed independent Student's *t*-test was applied to evaluate the statistical significance of differences between two groups. Results were considered statistically significant when *P*<0.05.

## SUPPLEMENTARY MATERIALS FIGURES AND TABLES


