# Supplementary material for: TRIB1 promotes colorectal cancer cell migration and invasion through activation MMP-2 via FAK/Src and ERK pathways
Source: Oncotarget. 2017 May 25;8(29):47931–42. doi: 10.18632/oncotarget.18201 (PMC5564616; doi:10.18632/oncotarget.18201)
Supplement: Supplementary file 1 [file oncotarget-08-47931-s001.pdf]

TRIB1 promotes colorectal cancer cell migration and invasion through activation MMP-2 via FAK/Src and ERK pathways

SUPPLEMENTARY MATERIALS

Supplementary Table 1: Correlation between TRIB1 expression and distant metastasis in CRC from GEO database (GSE17537)

Variable	Cases	TRIB1 expression		*P* value
		High	Low	
Distant metastasis				
-	38	24(63.2%)	14(36.8%)	0.002[a*]
+	17	3(17.6%)	14(82.4%)	

[a]Pearson χ2 test; [*]$P<0.05$.

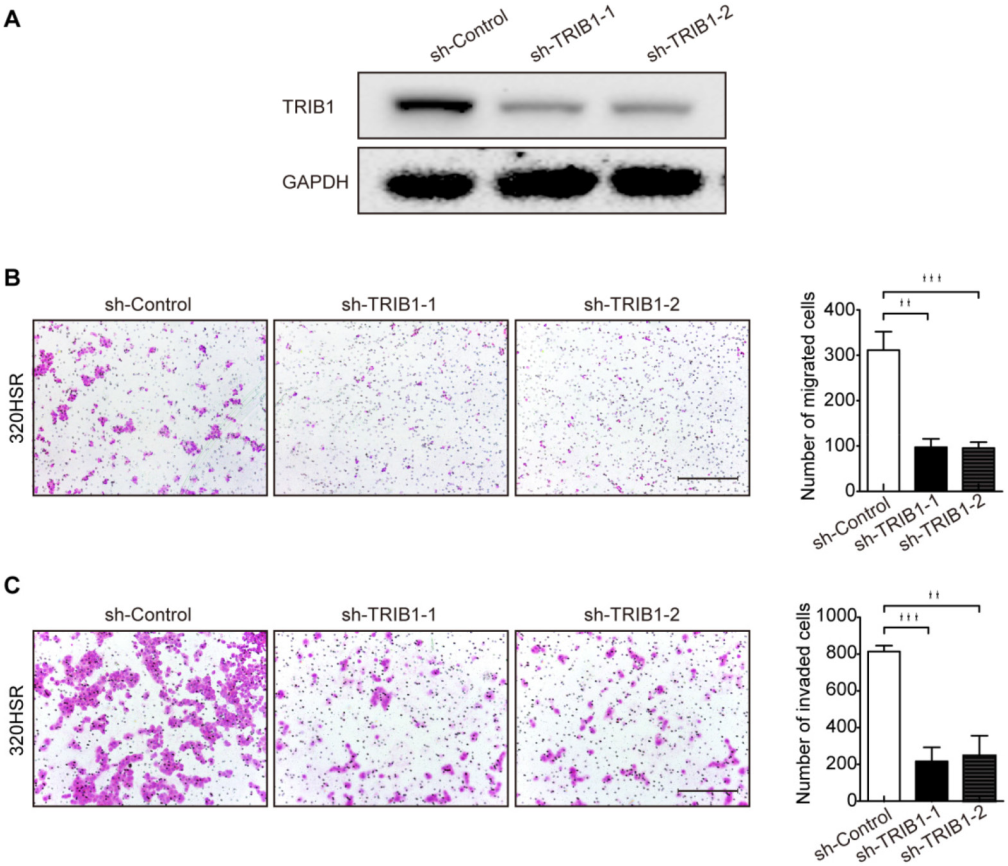

Supplementary Figure 1 (related to Figure 3): Silencing of TRIB1 reduces cell migration and invasion. (A) In COLO320HSR cells, the efficiency of sh-TRIB1-1 or sh-TRIB1-2 interference was confirmed by western blot analysis. Corresponding shRNA (sh-Control) was used as negative control and GAPDH was used as loading control. **(B)** Cell migration **(C)** and invasion were detected using transwell migration chamber and Matrigel invasion chamber, respectively. Examples of migrated or invaded cells are displayed in the left panels (magnification ×100). Scale bars = 200μm. Results are summarized as mean ± SD of triplicate experiments (**P*<0.01, independent Student's t-test) and shown in the right panels.
